# Cross-sectional study of patient-reported fatigue, physical activity and cardiovascular status in men after robotic-assisted radical prostatectomy

**DOI:** 10.1007/s00520-019-04794-1

**Published:** 2019-04-10

**Authors:** Ruth E. Ashton, Garry A. Tew, Wendy A. Robson, John M. Saxton, Jonathan J. Aning

**Affiliations:** 1 Department of Sport, Exercise and Rehabilitation, University of Northumbria at Newcastle, Newcastle upon Tyne, UK; 2 Department of Urology, Freeman Hospital, Newcastle upon Tyne, UK; 3 Bristol Urological Institute, North Bristol NHS Trust, Southmead Hospital, Bristol, UK

**Keywords:** Prostate cancer, Prostatectomy, Fatigue, Physical activity

## Abstract

**Purpose:**

Patient-reported fatigue after robotic-assisted radical prostatectomy (RARP) has not been characterised to date. Fatigue after other prostate cancer (PCa) treatments is known to impact on patient-reported quality of life. The aim of this study was to characterise fatigue, physical activity levels and cardiovascular status post-RARP.

**Methods:**

Between October 2016 and March 2017, men post-RARP or on androgen deprivation therapy (ADT) were invited into the study. Participants were asked to complete the Brief Fatigue Inventory (BFI) and Stage of Change and Scottish Physical Activity Questionnaires (SPAQ) over a 2-week period. Outcome measures were patient-reported fatigue, physical activity levels and the 10-year risk of cardiovascular disease (Q-Risk). Data were analysed in SPSS.

**Results:**

96/117 (82%) men approached consented to participate; of these, 62/96 (65%) returned complete questionnaire data (RARP *n* = 42, ADT *n* = 20). All men reported fatigue with 9/42 (21%) post-RARP reporting clinically significant fatigue. Physical activity did not correlate with fatigue. On average, both groups were overweight (BMI 27.0 ± 3.9 kg/m^2^ and 27.8 ± 12.3 kg/m^2^ for RARP and ADT, respectively) and the post-RARP group had an 18.1% ± 7.4% Q-Risk2 score.

**Conclusions:**

A proportion of men is at increased risk of cardiovascular disease within 10 years post-RARP and have substantial levels of fatigue; therefore, clinicians should consider including these factors when counselling patients about RARP. Additionally, men post-RARP did not meet the recommended guidelines for resistance-based exercise. Future research is needed to establish whether interventions including resistance-based exercise can improve health and fatigue levels in this population.

## Introduction

Radical prostatectomy is an accepted curative treatment option for men with clinically localised significant prostate cancer (PCa) with greater than 10 years of life expectancy and the ability to perform activities of daily living [1]. Robotic-assisted radical prostatectomy (RARP) is now the most prevalent modality for surgical removal of the prostate for PCa in the UK [2].

RARP has been demonstrated to be associated with lower blood loss and decreased hospital stay when compared to open radical prostatectomy [3]. Although commonly assumed that men undergoing RARP are fit and return to their pre-operative physical activity levels after surgery, there is little published data to substantiate this assumption. The prevalence of fatigue and post-operative physical fitness in men who have undergone RARP is largely unknown with few studies performed to date have explored cancer-related fatigue post-RARP. What data there is suggests that fatigue is present in PCa patients but is affected by treatment modality and the time period over which fatigue is assessed; however, it has been previously reported that approximately 14% of patients who have undergone radical prostatectomy experience fatigue [4, 5]. Fatigue in cancer patients and survivors has been associated with reduced physical activity levels [6], potentially adversely affecting cardiovascular risk profile and recovery to full functional fitness after RARP.

To our knowledge, no study has explored the association between self-reported physical activity, fatigue and comorbidities in men who have undergone RARP. The aim of this pilot study was to characterise fatigue, physical activity levels and cardiovascular status, over a 2-week period, in men after RARP and establish whether this is a substantial problem, which future intervention studies should address.

## Methods

### Design

A cross-sectional questionnaire study was administered to men who had undergone RARP and men treated with ADT for PCa. Men on continuous medical ADT, a treatment strongly associated with a number of side effects that impact quality of life including significant fatigue [7], were purposely used as a comparative population with which to relate the morbidity of RARP. The study was approved by the South East Scotland NHS Research Ethics Committee. Data from the ADT cohort of men is presented as a comparative control population.

This study was conducted at Newcastle upon Tyne Hospitals NHS Foundation Trust which is a tertiary referral centre serving a population of 1.2 million people. RARP was performed by three experienced surgeons at the institution over the study period.

### Participants

Men were eligible to participate in the study if they: (1) had histologically confirmed PCa, (2) were at least 8 weeks after their treatment for PCa with either RARP or after initiation of ADT, and (3) were able to provide consent and satisfactorily complete written questionnaires. All eligible patients attending outpatient’s clinics were approached. Men receiving any other treatment for PCa were excluded from the study.

### Study outcome data

Consenting men were asked to provide demographic information including current health status, average weekly alcohol intake and smoking status, and stature and body mass were measured. They were then invited to complete a questionnaire booklet containing validated questionnaires prospectively over a 2-week period (see further details below) and return the booklet in a prepaid stamp addressed envelope. Questionnaire score calculations were performed in accordance with published questionnaire protocols. Likewise, missing data were treated in accordance with the questionnaire protocols. The questionnaires included are detailed below.

#### Comorbidity and cardiovascular status

Charlson Comorbidity Index was calculated using information provided on stature, body mass and medical history [8, 9]. The risk of suffering a heart attack or stroke within the next 10 years was calculated using the validated objective measure: Q-Risk2 [10]. Q-Risk2 score is calculated from patient medical record data including family history, age, gender, ethnicity, socio-economic status, and selected physiological measurements, and can be categorised as <10% (low), 10%-20% (medium) or > 20% (high) [11]. Q-Risk2 was specifically used in this study, because in addition to being the NICE recommended formal risk assessment tool for CVD, it is also an accepted aid to clinical decision-making regarding how intensively to intervene to improve health in patients with CVD [12].

#### Scottish Physical Activity Questionnaire

The Scottish Physical Activity Questionnaire (SPAQ) was completed at the end of both weeks as a recall questionnaire and has good reliability (Cronbach’s alpha = 0.998) [13]. This questionnaire assesses moderate to vigorous physical activity (MVPA) over the previous 7 days. The questionnaire includes sections for both leisure time and occupational physical activity with each section containing questions on general activity such as walking, stair climbing and manual labour [13]. The average weekly total MVPA was calculated in addition to the mean total for each individual exercise component.

#### Brief Fatigue Inventory

The BFI was completed at the end of each day for all 14 days of the data collection period to rapidly assess fatigue in cancer patients and is correlated with other validated fatigue questionnaires [14–16] and has good reliability (Cronbach’s alpha = 0.95) [16]. The BFI consists of three questions assessing fatigue severity and six questions assessing the interference of fatigue with the patient’s mood and social/physical functioning with all answers being on a 0–10 scale. A global fatigue score was obtained for weeks 1 and 2 by averaging all the items on the BFI and as an average of the whole 2-week period [16]. Clinically significant fatigue is defined as a global fatigue score > 3 [5, 17].

#### Stage of Change Questionnaire

The Stage of Change Questionnaire was administered once at the start of the 2-week study period to assess patient’s attitudes towards exercise behaviour change and has acceptable reliability (Cronbach’s alpha = 0.63) [18]. Participants answered ‘yes’ or ‘no’ to four statements to assess each individual’s stage of behaviour change [6]. The stages are categorised as follows: stage 1—pre-contemplation, stage 2—contemplation, stage 3—preparation, stage 4—action and, and stage 5—maintenance.

### Statistical analysis

All returned surveys were included in the analysis, even if some sections were incomplete. Consequently, the number of total responses for each survey item varied because of missing data. Analyses were conducted using IBM SPSS Statistics Version 22 (IBM United Kingdom Limited, Hampshire, UK).

Normality was assessed using the Shapiro–Wilk and, if data was not normally distributed, transformations were conducted using common logarithms or square root. To assess the associations of the outcomes with self-reported total PA levels (SPAQ), Pearson correlations and Spearman’s rank were employed. Independent samples *t* tests were used to examine differences between the two treatment groups with *p* < 0.05 chosen as the accepted level of significance.

## Results

### Participants

In total, 148 men were approached to take part in the study and 96 men consented to participate in the study; of these, 62/96 (65%) patients returned postal questionnaires. Table 1 illustrates the demographic of the cohort. The patients approached were on average 11.7 months after RARP and 22.1 months after the initiation of ADT. The RARP cohort comprised 42/62 responses; of these, 57% and 14% were classified as overweight and obese, respectively.

**Table 1:** Patient demographics

	RARP (*n* = 42)	ADT (*n* = 20)
Age (years)	63.8 ± 6.4	67.3 ± 9.0
Body mass (kg)	86.7 ± 13.4	86.4 ± 12.3
Stature (cm)	180 ± 0.07	176 ± 0.07
Body mass index (kg/m^2^)	27.0 ± 3.9	27.8 ± 12.3
Drink alcohol *n* (%)	38 (90.5)	18 (80.0)
Months since treatment *mean* (range)	11.7 (2–115)	22.1 (2–120)
Pre-RARP PSA	10.05 ± 6.3	
Pathological Gleason Score (*n*)		
GS 6	2	
GS 3 + 4	25	
GS 4+ 3	7	
GS ≥ 8	8	
Pathological tumour stage (*n*)		
PT2	24	
PT3a	13	
PT3b	5	

Data are presented as mean ± standard deviation unless stated otherwise

*RARP* robot-assisted radical prostatectomy, *ADT* androgen deprivation therapy, *PSA* prostate specific androgen

### Cardiovascular status

Charlson Comorbidity Indexcalculations indicated that there was no significant difference in estimated 10-year survival after RARP (87.3% ± 12.2%) or ADT (80.5% ± 18.7%), *t*(27.2) = 1.5, *p* = 0.2. Q-Risk2 scores indicated that there was no significant difference in 10-year risk of suffering a heart attack or stroke between men post-RARP (18.1% ± 7.4%) and after initiation of ADT (22.4% ± 10.8%), *t*(28.4) = −1.6, *p* = 0.12.

### Physical activity

The levels of reported PA did not differ over the 2-week period between the two treatment groups (RARP total average mins = 658.1 ± 337.6 versus ADT total average mins = 631.9 ± 318.5, *t*(59) = 0.3, *p* = 0.8). Age, body mass, BMI and BFI scores were not associated with the total amount of PA performed in either treatment group (Table 2). Approximately 50% of all PA reported in both groups involved walking (e.g. walking to the shops/work, stair walking). Activities included in the ‘other’ category included yoga (1/42 post-RARP, 1/20 ADT), bowls (1/42 post-RARP) and rambling (2/42 post-RARP, 1/20 ADT). A breakdown of the amount of physical activity undertaken is illustrated in Table 3.

**Table 2:** Correlation matrix between physical activity and demographic factors, comorbidities, stage of change and fatigue

	Physical activityª	
	RARP	ADT
Age	− 0.1	− 0.14
Body mass	− 0.02	− 0.31
Body mass index	0.1	− 0.09
Stage of change	0.36^b^	0.15
Brief Fatigue Inventory	− 0.09	0.09

ªTotal physical activity in minutes averaged over the 2-week study period

^b^Correlation is significant at the 0.05 level

**Table 3:** Self-reported MVPA over the 2-week period

	RARP	ADT
Total (mins)	658.1 ± 337.6	631.9 ± 318.5
Walking (mins)	341.4 ± 245.5	319.5 ± 251.4
Manual labour (mins)	125.4 ± 168.3	92.3 ± 158.2
Active housework (mins)	57.6 ± 79.6	82.4 ± 89.4
Dancing (mins)	3.2 ± 11.3	0.63 ± 2.8
Sport/Leisure activities (mins)	92.3 ± 178.5	126.4 ± 209.4
Other activities (mins)	40.4 ± 100.2	6.3 ± 22.3

Data are presented as mean ± standard deviation

Two RARP participants did not provide physical activity data for week 1 or week 2

### Fatigue

**Fig. 1:**
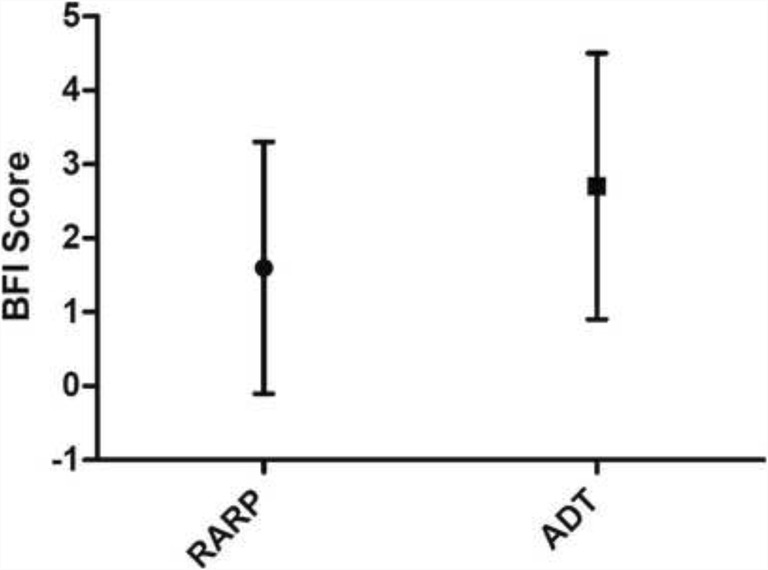
Brief Fatigue Inventory scores presented as a global score (range of scores 1–6) for RARP and ADT over the 2-week study period

All patients were experiencing fatigue over the 2-week study period; the majority of fatigue reported was mild-moderate in severity and of borderline clinical significance. The mean severity of fatigue was significantly less over the 2-week study period in the RARP (1.6 ± 1.7) than in the ADT group (2.6 ± 1.8), *t*(60) = − 2.628, *p* = 0.011 (Fig. 1). However, 9/42 (21.4%) patients’ post-RARP and 6/20 (30%) ADT patients reported clinically significant fatigue. There was no association between fatigue and the amount of self-reported PA (Table 2).

### Stage of change

The majority of patients in both treatment groups reported being in the maintenance stage of change (Table 4). The maintenance phase is where individuals have made specific modifications to their exercise behaviour; however, it requires a conscious effort to in order to maintain it. The stage of change outcomes positively correlated with the amount of PA undertaken in the RARP treatment group; this is shown in Table 2.

**Table 4:** Stage of change scores for both treatment groups

	RARP (*n* = 42)	ADT (*n* = 20)
Stage of change (number of patients)		
Stage 1—pre-contemplation	0	0
Stage 2—contemplation	1	3
Stage 3—preparation	0	1
Stage 4—action	5	3
Stage 5—maintenance	36	13

## Discussion

This is the first study to our knowledge to quantitatively explore cardiovascular risk, fatigue and physical activity, in men who have undergone RARP as a PCa treatment. Our study found that a substantial proportion of men post-RARP is at increased risk of suffering a cardiovascular related event within 10–15 years of their surgery and may suffer from clinically significant fatigue. Importantly, this study demonstrated that these findings were present in men appearing to meet the UK public health guidance of undertaking at least 150 min a week of moderate to vigorous aerobic physical activity. There was no significant difference between the cardiovascular risks found in men post-RARP and those on ADT in this study.

The present study identified that the Q-Risk2 score of the RARP cohort indicated that they were at a 18% greater risk of suffering a heart attack or stroke within the next 10 years. Whilst cardiovascular risk status has been studied extensively in patients receiving ADT [19–22], there are no studies, as far as the authors are aware to date, which have characterised the cardiovascular risk status of RARP patients. The largest most contemporary study by Wilt et al. [23] gives a signal as to mortality risk from causes other than PCa in a radical prostatectomy population. During a median follow-up of period of 10 years, 171/281 of the radical prostatectomy group died, and of these deaths, 74% (127/171) were not due to PCa [23]. Whilst it cannot be inferred that cardiovascular disease was the cause of all of these deaths due to other factors such as old age, this analysis combined with our study findings indicate potentially more can be done to improve the health of patients undergoing RARP. The Q-Risk2 calculator aids clinical decision-making about how intensively to recommend lifestyle interventions and lipid-lowering medications to patients with significant cardiovascular risks during patient-centred consultations [10]. The results of our study showed that our RARP cohort was at a 3% elevated risk of cardiovascular disease when compared to moderately active males of a similar age [24]. This finding, where it to be replicated in future studies, supports the view that men after RARP should at the very least be informed of their risk which could increase their compliance when offered lifestyle interventions to improve their cardiovascular health [25, 26].

Cancer-related fatigue has previously been reported as a side effect of PCa treatment in up to 80% of men [27–30]. Few studies have investigated levels of fatigue in men who have exclusively undergone RARP for PCa; however, much work has been conducted in men receiving ADT and radiotherapy. Storey et al. [5] performed a cross-sectional questionnaire study of recurrence free survivors who had undergone open radical prostatectomy utilising the BFI. Clinically relevant fatigue was identified in 22% (29/133) of men undergoing radical prostatectomy, whereas in their control non-cancer population, the incidence of clinically relevant fatigue was 16% (10/63) at a median follow-up of 56 months after treatment. Within their radical prostatectomy cohort median age 72, coexisting depression had the strongest independent association with fatigue. Storey et al. [5] did not examine PA levels within their cohort. Cancer-related fatigue has been acknowledged to be debilitating and to significantly impact on quality of life [31]. We have shown that after RARP in a contemporary younger population, similar to Storey et al., clinically relevant fatigue is reported by 20% of men at a mean follow-up of 11.7 months. This finding might be considered unexpected but highlights that post-treatment fatigue should be discussed with patients when they are counselled for RARP. All patients included in our study underwent holistic needs assessment after treatment and received targeted support if required as part of routine care from a survivorship nurse specialist [32, 33]. We have previously demonstrated that men in our institution who undergo RARP experience an unchanged overall quality of life [32]; therefore, it is probable that the clinically relevant fatigue identified in this study is unlikely to have resulted from psychological factors.

As far as the authors are aware, this is the first examination of PA sub-classifications undertaken in a contemporary population of men who have undergone RARP. Our study found patient-reported levels of PA after RARP which met current UK public health guidelines within the RARP cohort despite a high proportion of our patients having a high body mass index. We demonstrated that PA levels did not correlate with fatigue levels suggesting that fatigue levels may not be a barrier to the amount of PA undertaken within this population. Although public health guideline levels of aerobic PA were met, we identified that post-RARP patients did not achieve the recommended weekly amount of resistance exercise [34], with none of the patients reporting completing any resistance exercise. This important finding highlights a potential area of unmet need in the post-RARP population. Resistance exercise has previously been examined in other PCa treatment groups, both epidemiologically and during interventional studies which found resistance exercise to be safe in the population, alongside mitigating fatigue and generating longer-term improvements in quality of life, strength, triglycerides and body fat when compared to aerobic exercise [28, 35, 36]. The potential benefits of resistance exercise in relation to cardiometabolic risk profile were highlighted in a recent meta-analysis [37]. Although loss of skeletal muscle mass has been widely reported in PCa patients undergoing ADT [38–40] and many studies have investigated the impact of resistance exercise training programmes [35, 39, 41], much fewer studies have assessed changes in skeletal muscle characteristics after RARP. There is a need for future research to address this evidence gap, and extending the provision of structured exercise interventions (including resistance exercise) to this population may be warranted. Such interventions could have a positive impact on fatigue in men recovering from RARP, as demonstrated previously in fatigued PCa patients receiving ADT [29].

This study’s findings add quantitative depth to recent qualitative work performed by Sutton et al. [42] and Hackshaw-McGeagh et al. [43] identifying patients’ priorities. These studies showed that men undergoing RARP would value PA and dietary advice from their healthcare professional and would prefer to receive this at an early stage. In addition, they provided evidence that men undergoing RARP are willing to change their behaviour to improve their health, but they wish to be supported by their healthcare professional team to do so. Undergoing RARP is potentially a ‘teachable moment,’ and we have demonstrated that this population is at risk of both cardiovascular events and fatigue. Qualitative research shows men are receptive to health behaviour change [42–44], and such initiatives targeted at this population could have much potential to improve men’s overall health.

Our study supports consideration of further targeted research into strategies aimed at improving the health of men who have undergone RARP. Feasibility to recruit patients and compliance with completing study questionnaires has been demonstrated, in addition to the ability to discriminate the health status and behaviours of the RARP population. The present study has limitations. Whilst the patient-reported questionnaire data showed that all men appeared to meet UK guidance physical activity levels, the authors did not expect this finding. Additionally, we identified that the SPAQ does not allow for the separation of exercise at varying intensities, and therefore, potential over reporting of the amount of MVPA may take place. For example, within the walking category, some low intensity physical activity may have been included despite the instructions stating otherwise. We believe that our findings justify the inclusion of activity trackers used in parallel with patient-reported activity questionnaires in future study protocols to strengthen validity of activity outcome results. Our study did not investigate participants for sleep disorders. Although non-restorative sleep and fatigue are different entities, we acknowledge symptoms described by patients with each of these conditions may be similar and this should be investigated in future studies with a measure of sleep quality used alongside fatigue questionnaires [45]. In this study, we did not use a group of healthy men without cancer who had not undergone surgery as a comparator group and accept that this might be considered as a limitation. Patient and Public Involvement was integral to the design of this study. Prostate cancer patients felt that it was assumed that, because they were undergoing RARP, they were fit and that there was a lack of recommendations and guidance regarding fatigue, and health and lifestyle improvements they could make after RARP that should be addressed. This study was pragmatically designed in response to specific feedback from the Newcastle upon Tyne Patient and Public Involvement Group who felt that the comparator should be what they considered the most morbid prostate cancer treatment continuous ADT on the basis that, if similar morbidities were demonstrated, this would reinforce the need for targeted interventions in men after RARP. We acknowledge however that cardiovascular risk and fatigue will be present in the general healthy population. Finally, although the numbers included in this study were small, it has identified the need for further study in this population of men and informed the sample size calculation required for further work in this area. In order to conduct a fully powered study using the reported fatigue effect size from this pilot study (Cohen’s *d* = 0.57), the minimum total sample size to achieve 80% power (*α* = 0.05) was determined as *n* = 100 patients (50 patients in each group) would be needed to detect differences in fatigue between the two groups [46].

## Conclusion

Our study has shown that some men post-RARP are at increased risk of clinically significant consequences from cardiovascular disease within 10 years of their surgery and do suffer with clinically significant levels of fatigue. Clinicians should consider including these factors in the discussion when counselling patients about RARP. We have shown that men after RARP appear to meet the recommended guidelines for aerobic physical activity but do not meet them for resistance-based exercise. Future research is needed to establish whether exercise interventions can improve health and fatigue levels in this population.
